# Admission Grades as Predictors of Medical Students’ Academic Performance: A Cross-Sectional Study from Saudi Arabia

**DOI:** 10.3390/ejihpe12110110

**Published:** 2022-10-31

**Authors:** Ali Hendi, Mohammed S. Mahfouz, Ahmad Y. Alqassim, Anwar Makeen, Mohammed Somaili, Mohammed O. Shami, Abdellh A. Names, Alaa Darraj, Areej Kariri, Asma Ashiri, Abdulaziz H. Alhazmi

**Affiliations:** Faculty of Medicine, Jazan University, Jazan 45142, Saudi Arabia

**Keywords:** medical school, academic performance, admission criteria, predictors, MBBS, Jazan, Saudi Arabia

## Abstract

**Background**: Admission to medical school is competitive, and different countries use various tests in addition to high school grades to minimize selection bias. A few studies have been conducted to evaluate the usefulness of these tests as predictors for students’ academic performance. In this article, we aimed to assess factors that influenced students’ grades in medical school. **Methods**: A cross-sectional study included all students who graduated from the Faculty of Medicine at Jazan University between 2018 and 2020. Scores of the included participants were extracted from the registry of Jazan University, and additional questions about study habits were completed by the included students. Descriptive, univariate, and multivariate analyses were performed for the factors that impacted academic performance. **Results**: There were 331 included candidates, and the majority of them were female (53%). About 60% of the participants were medical residents at the time of the study, and 40% were interns. Univariate and multivariate analyses indicated that grades in high school and the pre-requisite tests were positively associated with students’ academic performance. Further, studying more than two hours per day was positively correlated with better grades in medical school. **Conclusion**: Scores of the admission tests can serve as predictors for student performance in medical school. National studies are deemed essential to evaluate additional admission tests for medical school, an action that would minimize selection bias.

## 1. Introduction

Most graduate programs stipulate that students meet certain basic academic requirements to be considered for admission. It is an accepted notion that, due to the marked variance in the quality and system of grading in high schools, it would be unfair if admission decisions were based solely on high school grades. As such, it is now a common practice in many countries (such as the United States of America and the United Kingdom) to have standardized tests for admission to universities and medical schools specifically [1,2,3]. This could take the form of either aptitude or achievement tests or both. While aptitude tests focus on assessing verbal and mathematical abilities [4], achievement tests are meant to measure the accomplishments of the candidates and are mostly based on certain guidelines and learnable concepts that the students are likely to learn in the future [5]. It was widely practiced that the grade point average (GPA) of the high school examinations was the sole basis for university admission, which led to variation in university admissions from school to school. This variation is attributed to various factors, such as the quality and type of education. Further, high school examinations may contain several subjects such as English, mathematics, and science subjects such as physics, chemistry, etc. Such heterogeneity prompted educational administrators to design a standardized admission test [6]. The benefits of standardized admission tests are obvious in that standardization and equal opportunity could be achieved [6].

In Saudi Arabia, there is strong competition for limited seats in medical schools [7]. This competition allows medical schools to be selective and admitted students are potentially able to complete their medical education and minimize the number of poor performers or ‘strugglers’, who were previously reported to account for 15% of students [7]. In 2004, the Admissions to Higher Education Steering Group in the United Kingdom warned against selection methods that were not valid and reliable, an attempt to reduce diversity in selection criteria among medical schools and to ensure an evidence-based approach [7,8]. Further, scores on standardized aptitude and achievement tests showed their predictive validity, in which students with higher scores are likely to have better academic performance in the university [9,10].

There is a dearth of information about the correlation between students’ entry eligibility tests and academic performance in the university in our region and to which extent those tests are reliable. Thus, the purpose of this study is to examine the correlation between entry qualifications and eligibility tests practiced in Saudi Arabia, i.e., general aptitude tests and academic achievement tests, and students’ academic performance in medical school. Further, in this study, we evaluate the relationship between the scores earned on the eligibility tests and the academic scores earned by the students.

## 2. Materials and Methods

### 2.1. Study Design and Participants

This was a retrospective cross-sectional study that was conducted between October and December 2021 at the Faculty of Medicine, Jazan University, which adopted an integrated organ-system curriculum conducted over seven years divided into four phases: a preparatory year, the pre-clerkship phase (during the second and third year), the clerkship phase (during the 4th, 5th, and 6th year) and finally the internship year. Jazan province is in southwestern Saudi Arabia and is populated by about 2 million inhabitants. The targeted students graduated between 2018 and 2020, which included all students who completed their six years of education.

### 2.2. Measures

An online self-administered questionnaire was distributed via students’ university emails to collect the demographic characteristics of the participants, and all other data were collected from the registry of students at Jazan university. Data included the following: high-school grades, aptitude test scores, achievement test scores, and the balanced percentage to determine their predictive value for academic performance as reflected by the college’s GPA.

### 2.3. Data Analysis

Statistical data entry and analysis were performed using SPSS v.23 (IBM Corp., Armonk, NY, USA). Data analyses involved descriptive statistics and also inferential statistics according to the required purpose of each relationship. Normally distributed data were managed by tests appropriate for this type of data. All categorical variables were presented as frequencies and percentages, while continuous variables were presented as means and standard deviations. Association between variables was investigated using the *t*-test or Chi-square test. A multiple linear regression model was applied to assess predictors of university academic performance. Statistical significance was set at a *p*-value < 0.05.

### 2.4. Ethical Clearance

The ethical approval to conduct the project was granted by the Jazan University Ethics Committee, with approval number REC-43/02/022, on 19 September 2021. We conducted this study following the ethical guidelines of the Helsinki Declaration and the local guidelines of the National Committee of Bioethics, Saudi Arabia. Participants in this study signed a consent to participate before data collection. All collected data were kept confidential and used for only the purpose of research. Additionally, the questionnaires did not include participants’ personal information or any other identification methods. All the participants were given the right to continue or withdraw at any time from the study.

## 3. Results

### 3.1. Sociodemographic Characteristics of the Study Participants

Table 1 shows a detailed description of the sociodemographic characteristic of respondents. A total of 331 participants agreed to participate in the survey. Among the 331 participants, 186 of them (56.2%) were females. Almost half of the participants lived in a village (*n* = 162, 48.9%), compared to 47.4% who lived in a city. Residents represented about 61%, while interns represented 39%. About 50% spent more than two hours daily studying.

### 3.2. Academic Performance

Table 2 and Table 3 summarize the differences between students’ performance during high school or university periods according to studied characteristics. No statistically significant differences were seen in all tests between males and females except for achievement tests and general aptitude tests, in which males recorded a better score compared to females (*p*-value = 0.009 and <0.001). Single and married students recorded a better performance in high school grades, first-year GPA, and cumulated GPA (CGPA) compared to divorced students (*p*-value = 0.034, 0.008, and 0.020), with no significant differences in their performance in other tests. Fathers’ occupations impacted student performance in achievement tests, and mothers’ occupations significantly impacted student performance in aptitude tests and first-year GPA. Additionally, studying hours per day seemed to impact high school scores and CGPA (*p*-value = 0.007 and 0.011). 

### 3.3. Correlations between Admission Grades and University Performance Variables

Table 4 shows a significant and positive correlation between aptitude exam grades and achievement test grades (Pearson’s r = 0.518, *p* < 0.01). Additionally, a significant and positive correlation between high school grades and achievement test grades was seen (Pearson’s r = 0.132, *p* < 0.05). A significant and positive correlation (*p* < 0.01) between high school grades (Pearson’s r = 0.231), aptitude exam grades (Pearson’s r = 0.315), achievement exam grades (Pearson’s r = 0.305) and graduation CGPA was also observed. Furthermore, a significant positive correlation (*p* < 0.01) was found between high school grades (Pearson’s r = 0.300), aptitude exam grades (Pearson’s r = 0.414), achievement exam grades (Pearson’s r = 0.407), graduation CGPA (Pearson’s r = 0.593) and first-year GPA. However, no significant correlation was recorded between high school grades and aptitude exam grades (Pearson’s r = 0.070, *p* > 0.05).

### 3.4. Multiple Linear Regression Models Assessing Predictors of University Academic Performance

Multiple linear regression analysis was performed (Table 5) to examine the adjusted associations between first-year GPA scores as well as graduation CGPA scores and the predictors included in the analysis. The analysis revealed that a one-degree increase in aptitude exam scores would increase the first-year GPA score (B = 0.033, *p* < 0.001). The analysis also revealed that a one-degree increase in achievement test scores would increase the GPA score (B = 0.028, *p* < 0.001). The analysis also revealed that a one-degree increase in high school scores would increase the GPA score (B = 0.150, *p* < 0.001). The analysis revealed that a one-degree increase in aptitude exam scores would increase the graduation CGPA score (B = 0.022, *p* < 0.001). The analysis also revealed that a one-degree increase in achievement test scores would increase the graduation CGPA score (B = 0.016, *p* = 0.004). The analysis also revealed that a one-degree increase in high school certificate scores would increase the graduation CGPA score (B = 0.086, *p* < 0.001). Additionally, the analysis revealed that an additional one hour spent studying would increase the graduation CGPA score (B = 0.102, *p* < 0.001).

## 4. Discussion

Various educational systems around the globe have adopted other cognitive and knowledge tests, in addition to high school scores, to minimize the potential selection bias in medical schools. In Saudi Arabia, two different tests were established to target graduates of secondary school who wish to pursue a degree in higher education. These two tests are the general aptitude test, which measures the analytical and deductive skills of students, and the academic achievement test, which evaluates knowledge of the basics of each subject they study [11]. The benefits of these exams were sought, and it was found that they are not limited to reducing selection bias; however, they are beneficial when used as predictive methods of students’ performance in medical school [1,2,3,6,7,8]. In Saudi Arabia, a few studies, mostly from the central region, were conducted to search for the correlation between admission exams and the academic performance of admitted medical students [7,12,13]. The current study was performed at Jazan University, in the southwestern region of Saudi Arabia, which serves about 2 million inhabitants with a capacity of about 150 to 200 spots each year for medical students to be enrolled [14]. The findings of our study suggest that the selection criteria are positively correlated with students’ academic performance (Table 2, Table 3 and Table 4), and these findings are similar to what others reported [7,12,13,15,16]. For example, Murshid observed a positive correlation between high school grades and achievement test scores and the GPA of medical students in a study conducted at Taibah University in 2012 [7]. Further, a similar observation of positive correlation was reported by Al-Qahtani and Alanzi in another study conducted at Imam Abdulrahman bin Faisal University between 2012 and 2013, which compared aptitude test scores, achievement test scores, and GPA scores of admitted students at medical and non-medical colleges [15]. These findings should encourage education officials to conduct a national study on medical schools for better evaluation of admission tests, and future improvements could be made accordingly based on the predicted results of the admitted medical students [17].

The international figure seems not different from what was reported in Saudi Arabia, in which admission tests are good predictors of students’ performance in medical school. For example, in a study conducted in Hamburg, Germany, between 2012 and 2015, students who recorded better academic performance in medical school were those who achieved better scores on admission tests [18]. Further, Meyer et al. observed that better performance in the entrance test was associated with the male gender [18]. In the current study, we observed that males performed better than females in both aptitude and achievement tests without a significant impact on their performance in medical school (Table 2). On the other hand, some countries added interviews as another factor to filter admitted students to medical school; this is practiced in the Goldman Medical School at Ben-Gurion University in Israel. Liberty et al. conducted a study and evaluated the performance of graduated interns in 2019 [19]. A similar positive association was observed between admission tests, including interviews, and the academic performance of candidates in medical school, and no significant difference between male and female performance was noted [19]. Another 5-year study from medical school at Universiti Brunei Darussalam reported that interviews, in addition to other admission tests, positively correlated with the academic performance of medical students [20]. Of note, interviews are not currently practiced as a selection criterion in our medical school despite being debated as an important non-cognitive test that may be considered for candidates to be admitted [21]. Thus, we believe that further studies are warranted to consider interviews for candidates with noticeable non-cognitive traits, which are crucial for physicians, and who have lower scores on admission tests [22,23].

Skills in time management have been repeatedly listed as one of the top factors in improving the academic achievement of medical students [24]. In the current study, we found students who spent a long time studying had better CGPA (Table 3). Interestingly, these skills of time management impacted scores in high school for those students (Table 2), indicating that these skills are acquired at an early age by the admitted students. Likewise, Alzahrani et al. reported a similar observation in a study conducted in 2015 at Taif University in Saudi Arabia [25]. Abdulrahman et al. conducted another study and found that spending more than two hours studying is associated with a higher GPA [26]. Ekwochi et al. performed a study in 2018 to evaluate the determinants of academic performance in medical students in Nigeria. They stated that better time management between extracurricular activities and devoted time for studying would likely result in better academic performance [27]. Shawwa et al. also shared the same observation in a study conducted at the Faculty of Medicine of King Abdulaziz University in October 2012 and found that students who spent more than 2 hours per day studying recorded a better GPA [28]. Taken together, time management skills are learnable, and the authors recommend medical schools teach students these skills to ensure better academic performance for medical students [24].

## 5. Limitations

Despite being one of the few studies in our region that evaluated predictive factors of medical students’ academic performance, this study bears many limitations. The nature of this study was conducted as a retrospective study, a method that has its known bias. Further, this study was conducted on graduates of three years (2018 to 2020); thus, it would be more interesting if this study were extended to include a larger population. However, we believe this study fills the gap of knowledge about some predictive tools for academic performance in medical schools in the southwestern region of Saudi Arabia and is to be followed by national studies for better exploitation of these tools.

## 6. Conclusions

The admission grades for medical school at Jazan University, including high school grades, general aptitude tests, achievement tests, and first-year GPA, showed their usefulness as a predictive tool for students’ academic performance. Further, time management skills impacted students’ GPAs. Future national studies are warranted to improve these predictive tools to minimize potential selection bias in a very competitive medical school.

## Figures and Tables

**Table 1:** Sociodemographic characteristics of the study participants (*n* = 331).

Variable		Number	%
Sex	Male	145	43.8%
	Female	186	56.2%
Age Groups (years)	23–24	52	15.7%
	25–26	191	57.7%
	27–31	88	26.6%
Location	City	157	47.4%
	Mountain	12	3.6%
	Village	162	48.9%
Social status	Single	243	73.4%
	Married	81	24.5%
	Divorced	7	2.1%
Parents’ marital status	Married, Living together	296	89.4%
	Married, non-living together	17	5.1%
	Divorced	18	5.4%
Studying hours per day	Less than 1 h	37	11.2%
	One Hour	48	14.5%
	Two Hours	80	24.2%
	More than 2 h	166	50.2%
Graduate status	Intern	130	39.3%
	Resident	201	60.7%

**Table 2:** Descriptive measures of the academic performance of the study participants in high school according to selected characteristics.

Variables		High School Grade				Achievement Exam Grade				Aptitude Exam Grade			
		Mean	SD	*p*-Value	t or F Tests	Mean	SD	*p*-Value	t or F Tests	Mean	SD	*p*-Value	t or F Tests
Gender	Male	99.0	1.2	*p* = 0.350	t = 0.870	80.7	6.3	*p* = 0.009 *	t = 6.930	85.8	5.1	*p* < 0.001 *	t = 6.930
	Female	99.1	1.3			79.0	5.8			80.5	6.0		
Location	City	99.1	1.4	*p* = 0.150	F = 1.910	79.3	6.0	*p* = 0.404	F = 0.908	82.6	6.3	*p* = 0.709	F = 0.344
	Mountain	98.4	1.3			79.4	5.3			83.8	5.8		
	Village	99.0	1.1			80.2	6.1			83.0	6.0		
Social status	Single	99.1	1.2	*p* = 0.034 *	F = 3.420	80.0	6.1	*p* = 0.392	F = 0.940	83.1	6.1	*p* = 0.249	F = 1.400
	Married	99.1	1.3			79.0	5.9			82.3	6.4		
	Divorced	97.9	1.4			78.6	6.8			79.7	4.3		
Father’s occupation	Business	98.6	1.1	*p* = 0.169	F = 1.620	78.2	7.4	*p* = 0.020 *	F = 2.950	81.5	5.6	*p* = 0.427	F = 0.965
	Government job	99.2	1.2			80.4	6.1			83.6	6.0		
	Private company job	98.8	1.5			78.8	7.2			80.0	7.2		
	Retired	99.0	1.3			79.9	5.7			82.6	6.3		
	Not working	99.6	0.3			75.0	6.9			82.1	5.9		
Mother’s occupation	Business	100	-	*p* = 0.208	F = 1.530	77.0	-	*p* = 0.214	F = 1.500	81.0	-	*p* = 0.037 *	F = 2.870
	Government job	99.3	0.9			81.1	5.8			84.8	5.8		
	Private company job	99.2	1.2			79.9	6.5			81.9	7.7		
	Retired	99.0	1.3			79.3	6.0			82.4	5.9		
Parents’ marital status	Married, Living together	99.1	1.2	*p* = 0.665	F = 0.408	79.7	6.2	*p* = 0.885	F = 0.157	82.8	6.3	*p* = 0.735	F = 0.308
	Married, Not living together	98.9	1.6			80.5	4.6			82.9	5.5		
	Divorced	98.9	1.4			79.7	4.6			83.9	5.1		
Studying hours per day	Less than 1 h	99.0	1.1	*p* = 0.007 *	F = 4.080	80.1	6.7	*p* = 0.935	F = 0.141	83.6	6.8	*p* = 0.464	F = 0.857
	One Hour	99.3	0.7			80.0	5.9			83.7	5.4		
	Two Hours	98.7	1.6			79.7	5.6			83.0	6.4		
	More than 2 h	99.2	1.1			79.5	6.2			82.4	6.1		
Intern/Resident	Intern	99.0	1.3	*p* = 0.480	t =0.500	80.6	6.0	*p* = 0.350	t = 4.500	83.6	6.0	*p* = 0.070	t = 3.314
	Resident	99.1	1.2			79.2	6.1			82.3	6.2		

* = Significant at *p* < 0.05.

**Table 3:** Descriptive measures of the academic performance of the study participants in medical school according to selected characteristics.

Variables		First-Year GPA				Graduation CGPA			
		Mean	SD	*p*-Value	t or F Tests	Mean	SD	*p*-Value	t or F Tests
Gender	Male	4.3	0.8	*p* = 0.449	t = 0.575	3.6	0.6	*p* = 0.218	t = 1.530
	Female	4.3	0.7			3.5	0.6		
Location	City	4.3	0.7	*p* = 0.602	F = 0.510	3.6	0.6	*p* = 0.904	F = 0.100
	Mountain	4.1	0.8			3.6	0.8		
	Village	4.3	0.7			3.5	0.6		
Social status	Single	4.4	0.6	*p* = 0.008 *	F = 4.910	3.6	0.6	*p* = 0.020 *	F = 3.980
	Married	4.1	1.0			3.4	0.6		
	Divorced	3.9	0.6			3.2	0.6		
Father’s occupation	Business	4.1	0.7	*p* = 0.543	F = 0.770	3.3	0.6	*p* = 0.363	F = 1.090
	Government job	4.4	0.7			3.6	0.5		
	Private company job	4.2	0.8			3.7	0.4		
	Retired	4.3	0.8			3.6	0.6		
	Not working	4.5	0.5			3.7	0.6		
Mother’s occupation	Business	4.9	-	*p* = 0.020 *	F = 3.330	4.3	-	*p* = 0.294	F = 1.243
	Government job	4.6	0.5			3.6	0.5		
	Private company job	4.3	0.8			3.5	0.6		
	Retired	4.2	0.8			3.5	0.6		
Parents’ marital status	Married, Living together	4.3	0.7	*p* = 0.865	F = 0.150	3.6	0.6	*p* = 0.876	F = 0.132
	Married, Not living together	4.4	0.6			3.6	0.5		
	Divorced	4.3	0.7			3.6	0.5		
Studying hours per day	Less than 1 h	4.0	1.0	*p* = 0.055	F = 2.570	3.3	0.6	*p* = 0.011 *	F = 3.800
	One Hour	4.4	0.7			3.5	0.6		
	Two Hours	4.3	0.6			3.5	0.6		
	More than 2 h	4.4	0.7			3.7	0.6		
Intern/Resident	Intern	4.4	0.7	*p* = 0.024 *	t = 5.130	3.7	0.6	*p* = 0.012 *	t = 6.330
	Resident	4.2	0.8			3.5	0.6		

* = Significant at *p* < 0.05. GPA = grade point average. CGPA = cumulative GPA.

**Table 4:** Correlation Matrix presenting Admission Grades and University performance variables.

Variables	High School Grade	Aptitude Exam Grade	Achievement Exam Grade	Graduation CGPA
Aptitude exam grade	0.070			
Achievement test grade	0.132 *	0.518 **		
Graduation CGPA	0.231 **	0.315 **	0.305 **	
First-year GPA	0.300 **	0.414 **	0.407 **	0.593 **

The bivariate correlation is based on Pearson’s correlation coefficient. * = significant at *p* < 0.05, ** = significant at *p* < 0.001. GPA = grade point average. CGPA = cumulative GPA.

**Table 5:** Multiple Linear regression models assessing predictors of university academic performance.

Model 1: Outcome = First-Year GPA								
Factors	Unstandardized Coefficients		t	*p*-Value	95% CI		F Value	*p*-Value
	β	SE			Lower	Upper		
(Constant)	−15.561	2.790	−5.577	<0.001	−21.049	−10.072	43.81	<0.001
Aptitude exam grade	0.033	0.007	5.077	<0.001	0.020	0.046		
Achievement test grade	0.028	0.007	4.175	<0.001	0.015	0.041		
High school certificate grade	0.150	0.028	5.307	<0.001	0.094	0.206	R^2^ = 0.28	
**Model 1: Outcome = Graduation CGPA**								
**Factors**	**Unstandardized Coefficients**		**t**	***p-*** **value**	**95% CI**		**F value**	***p-*** **value**
	**β**	**SE**			**Lower**	**Upper**		
(Constant)	−8.353	2.313	−3.612	<0.001	−12.903	−3.804	19.9	<0.001
Aptitude exam grade	0.022	0.005	3.972	<0.001	0.011	0.032		
Achievement test grade	0.016	0.006	2.865	0.004	0.005	0.027		
High school certificate grade	0.086	0.023	3.664	<0.001	0.040	0.132	R^2^ = 0.20	
Studying hours per day	0.102	0.028	3.677	<0.001	0.048	0.157		

GPA = grade point average. CGPA = cumulative GPA. CI = confidence interval. SE = standard error.

## Data Availability

Data are available upon request from the researchers.
